# A skin-specific α-Synuclein seeding amplification assay for diagnosing Parkinson’s disease

**DOI:** 10.1038/s41531-024-00738-7

**Published:** 2024-07-04

**Authors:** Yaoyun Kuang, Hengxu Mao, Tingting Gan, Wenyuan Guo, Wei Dai, Weimeng Huang, Zhuohua Wu, Hongyan Li, Xiaoyun Huang, Xinling Yang, Ping-Yi Xu

**Affiliations:** 1 Department of Neurology, the First Affiliated Hospital of Guangzhou Medical University, Guangzhou, 510120 China; 2 Department of Neurology, the First Affiliated Hospital of Gannan Medical University, 341000 Ganzhou, China; 3 Department of Neurology, Xinjiang Uygur Autonomous Region People’s Hospital, 830054 Urumqi, Xinjiang China; 4 Dongguan Songshan Lake Central Hospital, 523000 Donggguan, China; 5 The Second Affiliated Hospital of Xinjiang Medical University, 830054 Urumqi, Xinjiang China

**Keywords:** Diagnostic markers, Predictive markers

## Abstract

The seeding amplification assay (SAA) has recently emerged as a valuable tool for detecting α-synuclein (αSyn) aggregates in various clinically accessible biospecimens. Despite its efficiency and specificity, optimal tissue-specific conditions for distinguishing Parkinson’s disease (PD) from non-PD outside the brain remain underexplored. This study systematically evaluated 150 reaction conditions to identify the one with the highest discriminatory potential between PD and non-synucleinopathy controls using skin samples, resulting in a modified SAA. The streamlined SAA achieved an overall sensitivity of 92.46% and specificity of 93.33% on biopsy skin samples from 332 PD patients and 285 controls within 24 h. Inter-laboratory reproducibility demonstrated a Cohen’s kappa value of 0.87 (95% CI 0.69–1.00), indicating nearly perfect agreement. Additionally, αSyn seeds in the skin were stable at −80 °C but were vulnerable to short-term exposure to non-ultra-low temperatures and grinding. This study thoroughly investigated procedures for sample preprocessing, seed amplification, and storage, introducing a well-structured experimental framework for PD diagnosis using skin samples.

## Introduction

Definitive diagnosis of PD relies on postmortem identification of disease‐associated α‐synuclein (αSynD), characterized by the presence of misfolded protein aggregates in the central nervous system (CNS), a clinical acquisition that proves exceptionally challenging^1^. Substantial evidence supports the notion that pathological αSyn aggregates exhibit prion-like characteristics in recruiting and converting free monomers and are capable of undergoing cell-to-cell transmission between the central and peripheral regions^2–4^. This unveils the opportunity to identify αSynD beyond the central nervous system (CNS). Development of a reliable and sensitive assay for detecting extracranial pathological αSyn aggregates can, to a certain extent, reflect the deposition level of central αSynD^5^. Consequently, SAA was designed to facilitate the exponential replication of pathological αSyn in vitro, allowing for the effective detection of traces of αSyn aggregates in biological samples with high sensitivity and specificity^6,7^. The utilization of in vitro amplification systems for the detection of αSynD in the skin as an easily accessible extracranial sample, represents an innovative approach for pre-mortem diagnosis. Previous studies have shown the diagnostic potential of SAA by successfully amplifying αSynD from synucleinopathies utilizing skin samples^8–12^. Despite the initial findings, establishing SAA as a routine diagnostic test in clinical practice necessitates thorough validation and standardization. Few studies address the complete process to standardize the entire procedure, encompassing sample pretreatment, storage, and tissue-specific αSynD amplification in PD. These factors have the potential to artificially decrease or increase αSyn seeding activity, leading to false positive or negative results. Hence, we systematically evaluated 150 distinct reaction conditions, incorporating various thermal conditions, monomeric species, and different additives seeded with fully characterized skin homogenates from a PD patient and a healthy control(HC). The outcome of this evaluation led to the development of a thermal-dependent (50 °C) amplification assay, ensuring the detection of αSynD with sensitivity, specificity, and reproducibility.

## Results

### Optimizing conditions for SAA to screen for αSyn seeding in PD skin

**Fig. 1:**
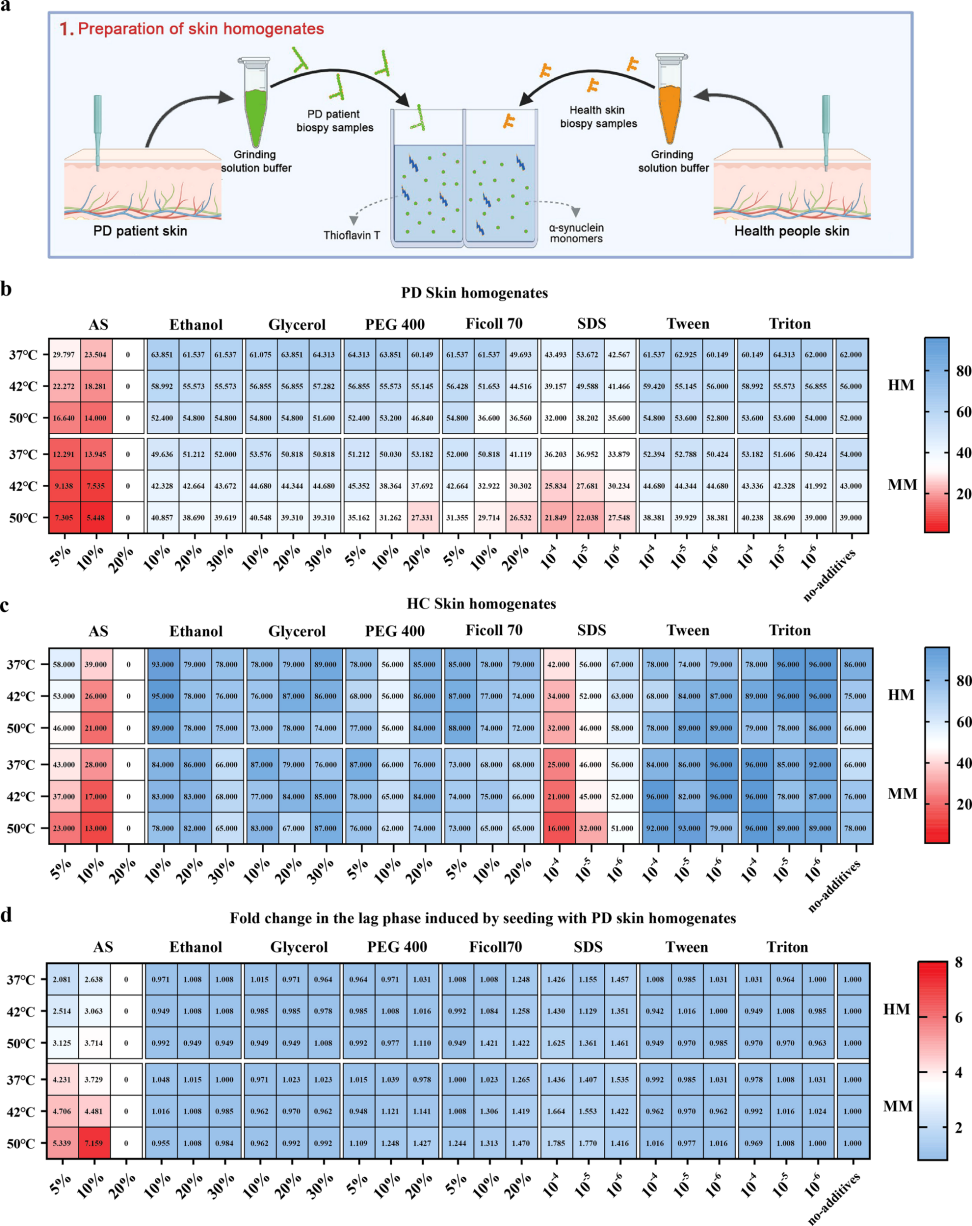
Changes in SAA physicochemical factors promote the detection of αSyn seeding in PD. **a** Schematic of the initial phase in the seeding amplification assay (SAA) buffer discovery process for assessing α-synuclein (αSyn) seeding in Parkinson’s Disease (PD). In Step 1, skin samples were extracted from both PD patients and HC. In Step 2, these samples underwent incubation with 126 distinct conditions, followed by cycles of agitation and rest for evaluation. Designed with Biorender.com. **b** and **c** Aggregation rate between reactions seeded with PD‐derived skin homogenate and control samples were amplified under two different monomeric species using 7 additives at 3 concentrations in 3 different temperatures, with no addition group as control. **b** displays the outcomes of SAA seeded with skin homogenate from PD and **c** displays the outcomes of SAA seeded with skin homogenate from HC. The lag phase was calculated as the mean of the values obtained from each triplicate. **d** A favorable environment to detect disease‐associated α‐synuclein (αSynD) in PD skin was shown in the fold separation of the lag phase between PD and HC. Buffers lighted in red represent the optimal conditions to detect αSynD seeding in PD skin.

The pathological αSyn typically exists in an aggregated form, serving as seeds to autonomously recruit soluble monomers and replicate themselves^13,14^. It has been reported that external conditions, like temperature, shaking cycles, monomeric species, protein concentrations, pH, ionic strength, etc., were key parameters for the fibrillation of prion-like proteins^15,16^. Moreover, it has been noted that brain homogenate and cerebrospinal fluid (CSF) from both HC and synucleinopathy cases exhibit an inhibitory effect on αSyn aggregation when compared to the use of the buffer alone^7,17,18^. This suggests that both sample properties and amplification conditions collectively influence the outcomes of the experiments. To validate the hypothesis that altering the physicochemical factors influencing the in vitro amplification of amyloidogenic proteins would enhance αSynD seeding in a tissue-specific manner, we systematically evaluated 150 diverse reaction conditions using an array of different thermal conditions, monomeric species and additives, seeded with fully characterized skin homogenates from PD patients and HC (Fig. 1a). We compared the αSyn SAA in the presence of hydrated anions (SO_4_^−^), molecular crowding agent (PEG 400 and Ficoll) at high (20%), medium (10%) and low (5%) concentrations and Ethanol, and Glycerol at high (30%), medium (20%) and low (10%) concentrations, with no addition group as control. We also tested the effects of SDS, Triton, or Tween on skin-seeded reaction mixtures at high (0.01%), medium (0.001%), and low (0.0001%) concentrations using mouse αSyn monomer (MM) or human αSyn monomer (HM) in three thermal conditions (37, 42, 50 °C). In the resulting 150 reaction conditions, equal amounts of skin homogenate were used per reaction for three different replicates. The same amounts of skin homogenate from HC were also added in each experimental condition. The kinetic parameters and lag phase (which correspond to the time needed to reach the threshold) were used for measuring the rate of seeding activity at each condition. Figure 1b revealed that 144/150 reaction conditions were able to detect αSyn aggregates, showing a lag phase between 5.44 and 64.3 h; 97/144 condition showed no major differences in the kinetic curve of the reaction condition when the skin homogenate from PD patients was presented in the buffer condition as compared to the no-addition group at indicated temperature. From these buffers, the conditions (0.1 mg/ml MM, 10% w/v ammonium sulfate (AS), 10 mM ThT, 50 mM NaCl, 10 mM Tris–HCl, pH 7.5.) were amplified at 50 °C that displayed the shortest lag phase. The results of SAA seeded with skin homogenate from HC are shown in Fig. 1c. It showed a substantial disparity in the lag results between individuals with PD and HC under the optimal SAA condition. The fold separation of the lag phase is calculated in the PD group by dividing the lag phase obtained from each condition by those from the HC group under the same condition (Fig. 1d). Under the streamlined SAA condition, there was a seven-fold increase in speed compared to the no-additive group. The aforementioned optimal SAA condition was then selected for further analysis.

We also tested the sensitivity of the aforementioned buffer condition using the gradient dilution of human pre-formed fibrils (hPFFs), the results showed the artificial seeds could be detected after 10^−7^ dilutions in 12 h, while no aggregates were seen in the group without hPFFs. It indicated that the sensitivity of the streamlined protocol reached 100 fg (Supplementary Fig. 1a). To investigate if there was a cross-seeding effect in the buffer condition, the PFFs of another amyloid protein—Tau (ζ306 (2R), K19CFh (3R)) were used to seed monomeric mouse-αSyn. Consistently, no signal was detected in the presence of tau seeds, even when the concentration of Tau PFFs was as high as 0.1 mg/ml. This result confirms there is no cross-seeding between αSyn and Tau amyloidogenic proteins and confirms the specificity of the protocol (Supplementary Fig. 1b).

### Detection of αSyn seeding activity in biopsy skin samples from PD patients

**Fig. 2:**
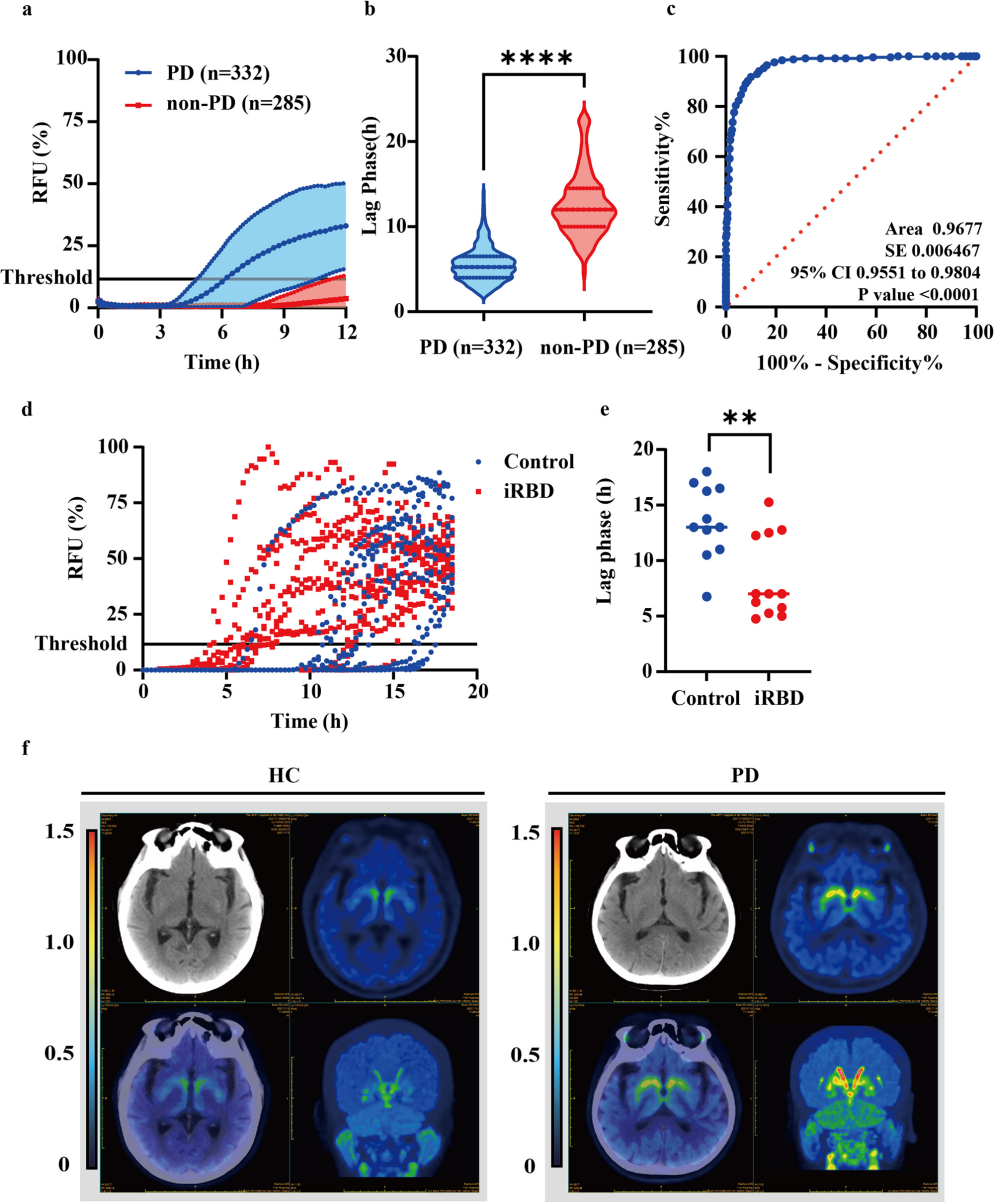
Detection of αSyn seeding activity using the modified SAA in biopsy skin samples from PD patients. **a** Modified SAA was used to detect αSyn seeding activity in the skin homogenates from 332 patients with PD and 285 non-PD controls. **b** Comparison of the lag phase of the modified SAA between PD patients and non-PD controls. Data are presented as mean ± SD of all patients analyzed in each group. **c** Receiver operating characteristic (ROC) curve and the corresponding area under the curve (AUC) for the lag phase. ROC analysis demonstrates an optimal threshold of 8.375 h. **d** ThT curves of αSyn seeding activity from patients with HC (blue, *n* = 11) and isolated rapid-eye-movement sleep behavior disorder (iRBD) (red, *n* = 12) utilizing skin samples. **e** Comparison of the lag phase between patients with iRBD and controls. Data are presented as mean ± SD of all patients analyzed in each group. **f** Representative PET-CT image of vesicular monoamine transporter 2 (VMAT2) distribution in PD (*n* = 20) and HC (*n* = 8). The two-tailed independent Student’s *t*-test was used in **b** and **d**, data are shown as mean ± SD. Data are statistically different from each other with ***P < 0.001.

To determine whether the above findings can be applied to a large cohort of samples with PD, skin obtained by punch biopsy from posterior cervical sites was examined using the streamlined SAA strategy. In total, 332 skin samples obtained from individuals clinically diagnosed with PD were utilized, with an additional 285 samples collected from healthy individuals or patients with other neurological diseases serving as controls. Figure 2a shows the ThT kinetics of aggregation for all samples from the PD and controls after 12 h of incubation. The average lag phase was significantly shorter in PD samples than in controls; the lag phase of the samples from PD patients was approximately 5.5 ± 2.0 h, whereas αSyn aggregation in the presence of skin from control samples was 12.5 ± 3.7 h (Fig. 2b). The overall differences between samples from patients with PD and controls were significant (*P* < 0.001). A post hoc receiver operating characteristic (ROC) analysis revealed an optimal skin SAA cut-off at 8.375 showing a maximum of 90.2% specificity and 91.2% sensitivity (Fig. 2c). Based on the cut-off of the skin SAA, 307 out of 332 (92.46%) were deemed positive. Conversely, of the 285 unrelated disease controls tested, 266 (93.33%) were correctly scored as negative by αSyn seeding (Table 1).

**Table 1:** Characteristics of the study participants and the streamlined αSyn SAA results in skin samples

	PD (*n* = 332)	Non-synucleinopathy controls (*n* = 285)
Age, mean (SD)	65.2 (10.1)	60.7 (9.9)
Sex, male (female)	201 (131)	140 (145)
Duration of disease	5.0 (3.78)	NA
Hoehn and Yahr scale	2.8 (0.93)	NA
UPDRS III	34.5 (21.42)	NA
streamlined SAA positive	307	19
streamlined SAA negative	25	266
Sensitivity	92.46%	
Specificity	93.33%	

PD, Parkinson’s disease; NA, not applicable; SAA, Seeding amplification assay.

To investigate the distinction between SAA and IF in detecting pathological αSyn, we randomly selected 12 SAA-positive PD and 8 SAA-negative controls, and IF has been implemented. Analysis of sensitivity and specificity showed that p-αSyn presented a high diagnostic accuracy in discriminating PD from non-synucleinopathies with 91.7% sensitivity and 100% specificity (Supplementary Table 1). The skin SAA assay showed an agreement with the p-αSyn staining in skin disclosed by immunofluorescence (*κ* = 0.898; *p* < 0.001), which would provide valuable insights into the correlation and potential differences between the two techniques.

It is worth noting that 12 patients with clinically diagnosed isolated rapid-eye-movement (REM) sleep behavior disorder (iRBD) and 13 HC were also recruited into our trial. iRBD can be part of the prodromal stage of synucleinopathies^19,20^. Previous work from other groups has shown that detection of misfolded αSyn in patients with iRBD was associated with an increased risk of subsequent diagnosis of PD, multiple system atrophy (MSA), or dementia with Lewy bodies (DLB), which represents a potential prodromal marker of these diseases^20,21^. Figure 2d shows the ThT kinetics of aggregation of all the samples from the iRBD and control groups within 24 hours of incubation. The results were considered positive in 8 out of 12 (66.6%) patients with iRBD, while 11 out of 12 (91.6%) HC were deemed negative (Fig. 2e). Our findings indicate that this approach has the ability to detect αSyn in patients with iRBD, enabling identification of patients that may have a higher risk of developing clinically defined synucleinopathy than patients with iRBD who are αSyn negative.

To further validate and confirm the accuracy of the modified SAA results, we randomly selected 20 αSyn SAA-positive PD patients and 8 non-neurodegenerative controls who underwent PET-CT AV133 scanning. The results confirmed an asymmetric decrease in dopamine metabolism in the striatum of all the PD patients with an overall accuracy of 100%, while no such decrease was observed in the HC group (Fig. 2f), supporting the sensitivity and specificity of the assay for PD diagnosis.

### Grinding, but not high-temperature pretreatment, affected the seeding activity of αSynD

**Fig. 3:**
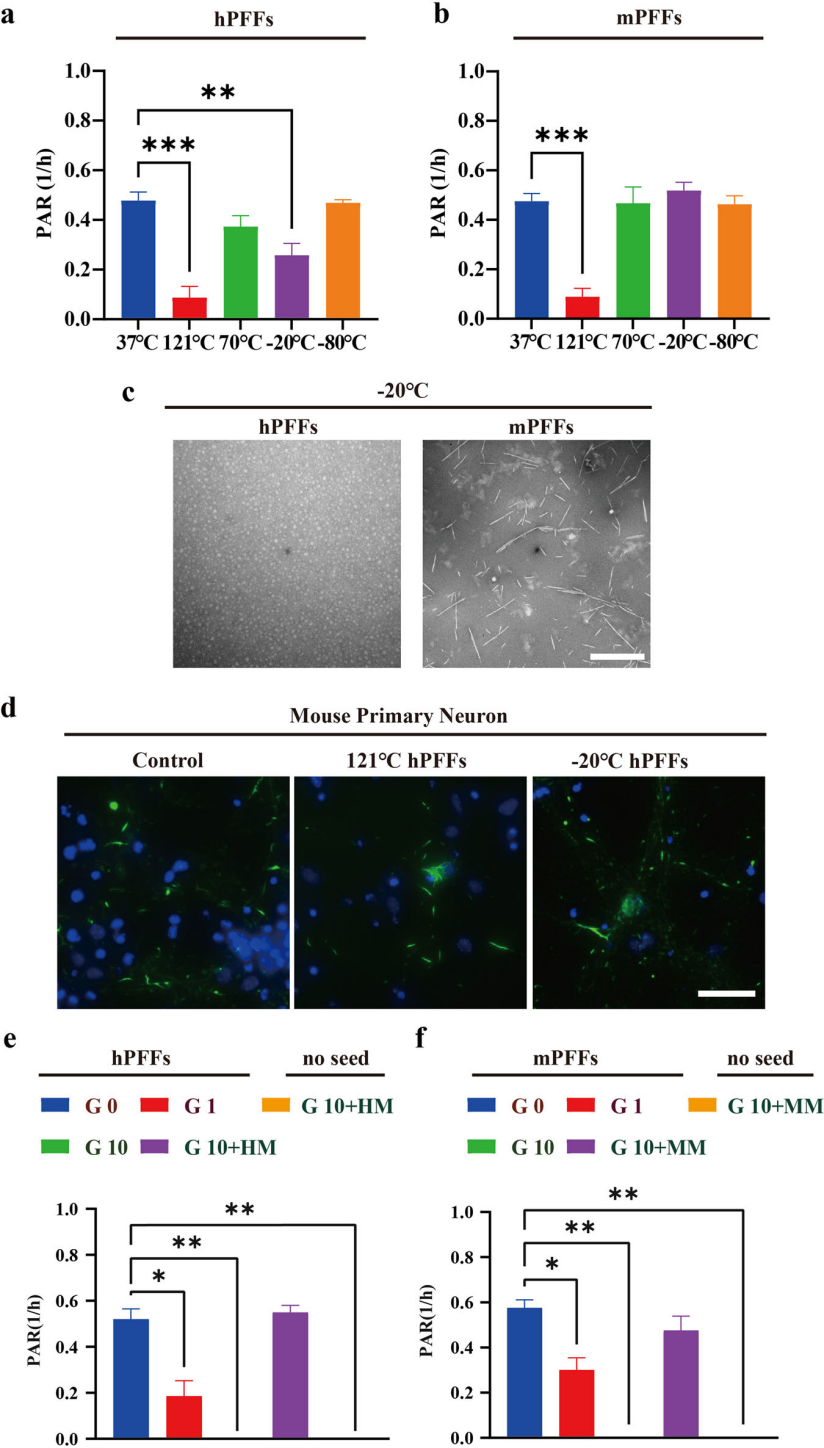
Effects of pretreatments on seeding ability of hPFFs/mPFFs. **a** and **b** Effect of pretreatment on varied temperature on the seeding ability of human pre-formed fibrils (hPFFs)/ mouse pre-formed fibrils (mPFFs). 1 μg/ml hPFFs/mPFFs were exposed to heating (121, 70 °C) or cooling (−20, −80 °C) for 1 h before being utilized in the streamlined SAA. **c** Transmission electron microscopy (TEM) images of hPFFs/mPFFs after cooling pretreatment at −20 °C for 72 h. The hPFFs depolymerized into 20 nm diameter oligomers, while the mPFFs showed no significant depolymerization compared to hPFFs. Scale bar = 200 nm. **d** Immunofluorescence image of pS129-Syn in primary neurons exposed to heating (121 °C) or cooling (−20 °C) pretreated hPFFs (1 μg/ml) for ten days, untreated hPFFs were used as control. Scale bar = 25 μm. **e** and **f** Effect of grinding pretreatment on the seeding ability of hPFFs/mPFFs. 1 μg/ml hPFFs/mPFFs were pretreated by grinding for either 0, 1, or 10 times before being utilized in SAA. The decreased seeding capacity from over-grinding (10 times) was reversed by the extra addition of 0.1 mg/ml of homologous monomer to the grinding solution. One-way ANOVA followed by Tukey’s post-hoc-test was used, data are shown as mean ± SD. Data are statistically different from each other with *P < 0.05, **P < 0.01, and ***P < 0.001.

To explore the influence of sample pretreatment on the seeding activity of αSynD, the effect of heating and cooling pretreatment on the seeding activity of αSynD through the streamlined amplification protocol was examined, since αSyn is extremely heat-resistant and low temperature might induce protein depolymerization which can affect the preservation of samples^22,23^. For this purpose, hPFFs and mouse pre-formed fibrils (mPFFs) were used to simulate αSyn aggregates in samples, and different degrees of heating (121 °C, 70 °C) and cooling (−20 °C, −80 °C) were performed before these PFFs were amplified in the system, with untreated PFFs as control. As illustrated in Fig. 3a, b, the heating pretreatment has a negligible impact on the seeding activity of αSynD, hPFFs, and mPFFs could still promote αSyn fibrillation after 70 °C pretreatments for one hour, as evidenced by the unchanged aggregation rate of both hPFFs and mPFFs. However, pretreatments at 121 °C significantly reduce the aggregation rate of αSyn compared to the untreated group. Regarding cooling pretreatment, even though exposing hPFFs at −20 °C for one hour leads to the depolymerization of hPFFs but not mPFFs into 20 nm diameter oligomers (Fig. 3c), these hPFFs were still partially able to promote αSyn fibrillation as shown by retained PAR (Fig. 3a, b). Storing sonicated h/mPFFs at −80 °C for an hour also preserves their seeding activity, indicating that neither heating nor cooling alone can eliminate the seeding ability of both hPFFs and mPFFs after a brief exposure. To further verify this hypothesis, mouse primary neurons were exposed to 121 or −20 °C pretreated hPFFs (1 μg/ml). Consistently, we could still observe the formation of pS129 αSyn in primary neurons ten days post-treatment (Fig. 3d), indicating short-term heating or cooling is not an effective way to inactivate αSynD. In addition to temperature, the effect of grinding, which provided a theoretical basis for the sample preparation was further investigated. The results revealed that a single cycle of grinding with zirconia beads did not diminish the seeding capability of both hPFFs and mPFFs as indicated by an unchanged aggregation rate compared to the untreated group. Nevertheless, the fibrillation of αSyn diminished with an increase in grinding cycles, and αSyn seeding activity nearly vanished following 10 cycles of grinding (Fig. 3e, f). To mitigate the decrease in seeding activity resulting from grinding, we added 0.1 mg/ml of monomeric αSyn to the h/mPFFs before the grinding process, with the same amount of monomeric αSyn as a control. Importantly, introducing monomeric αSyn into the buffer prior to grinding reversed the decline in h/mPFFs’ seeding capacity caused by grinding. The control group, without the addition of αSyn PFFs, did not exhibit any seeding capability. This hints at a possible strategy: when handling items with minimal amounts of αSynD, incorporating a small amount of αSyn monomers might mitigate the effects of grinding on their seeding activity.

### Evaluating the seeding response in skin samples across different storage conditions and plates using the optimized SAA

**Fig. 4:**
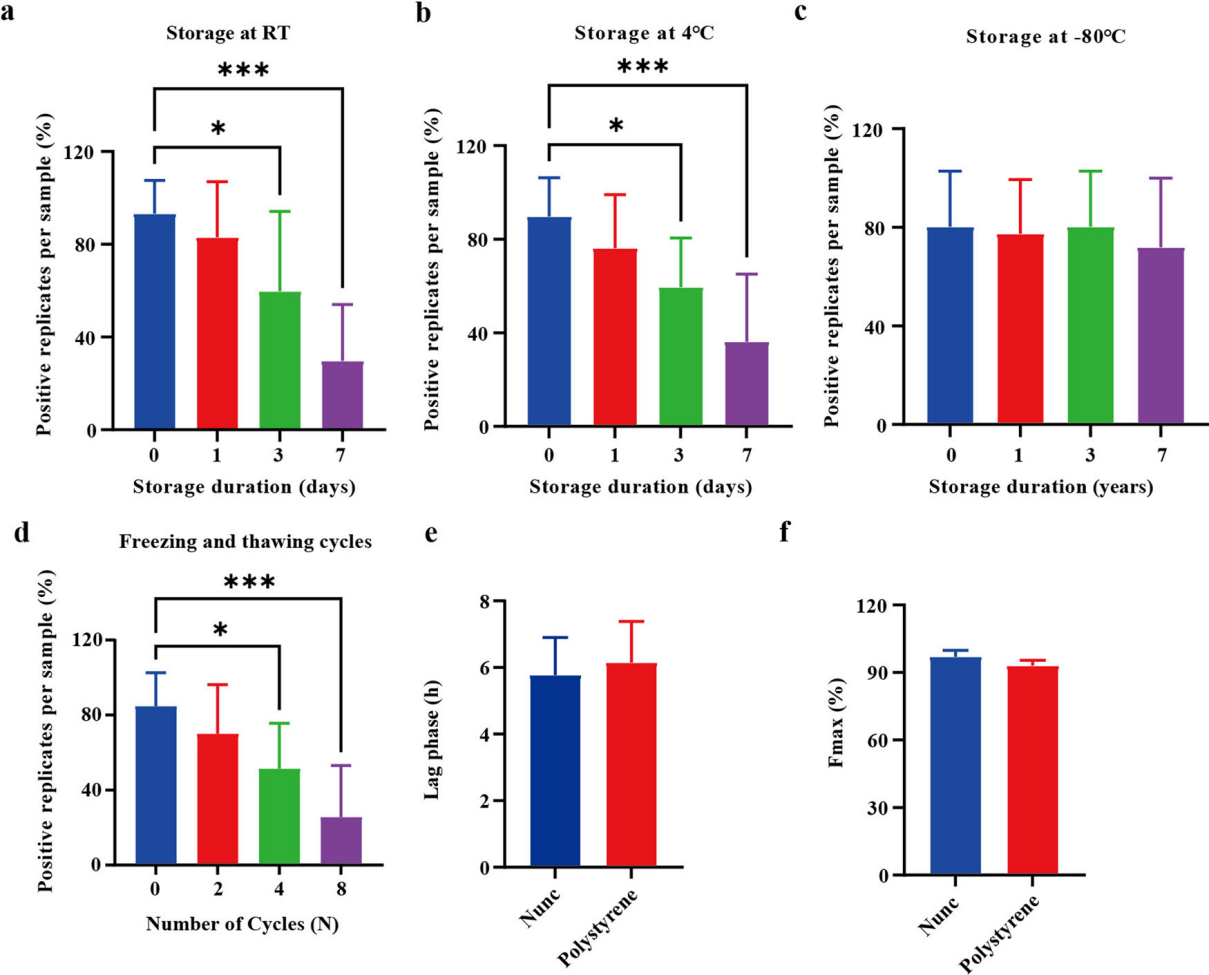
Effect of various storage conditions on the seeding activities of skin-derived αSynD. **a** and **b** Streamlined SAA reactions seeded with skin homogenate from PD patients were analyzed under short-term storage conditions (0, 1, 3, and 7 days) at room temperature (RT) and 4 °C. **c** Streamlined SAA reactions seeded with skin homogenate from PD patients were analyzed after long-term storage (up to 3 years). **d** Streamlined SAA reactions seeded with skin homogenate from PD patients were analyzed after repeated freezing and thawing cycles (0, 2, 4, and 8 cycles). Numbers of positive replicates were calculated for each sample in percent. **e** and **f** Comparison of the lag phase and Fmax of the modified SAA between Nunc and polystyrene plates. Data are presented as mean ± SD of all PD patients analyzed in each group. One-way ANOVA followed by Tukey’s post-hoc-test was used in **a–d**, the two-tailed independent Student’s *t*-test was used in **e** and **f**, data are shown as mean ± SD. Data are statistically different from each other with *P < 0.05 and ***P < 0.001.

Skin homogenates obtained from 10 individuals with PD and 8 controls were placed in polypropylene tubes and stored at either room temperature (RT) or 4 °C for varying durations (0, 1, 3, and 7 days). The study also examined the impact of extended storage (1–3 years in a −80 °C freezer) on the stability of αSyn seeding response in 12 PD patients and 7 controls. Additionally, skin samples from 9 PD patients and 7 controls underwent multiple freezing and thawing cycles (0, 2, 4, and 8 cycles). For each indicated condition, the percentage of positive replicates per sample was calculated (3/3 = 100%, 2/3 = 66%, 1/3 = 33%, and 0/3 = 0%), which is crucial for assessing the seeding activity of the samples. The results indicated under short-term storage conditions, the αSyn seeding ability starts to decline at day 3, whether kept at RT or 4 °C. Beyond this period, there is a notable decline in the percent of positive replicates at day 7 (Fig. 4a, b). The seeding ability of αSyn remained stable under the analyzed long-term storage conditions for up to 3 years (Fig. 4c) and was resistant to up to 4 repeated freezing and thawing cycles (Fig. 4d). All control samples without synucleinopathy showed negative SAA responses under the above storage conditions (data not shown), suggesting that specificity remains constant under these conditions. These results indicate that αSyn seeds become unstable in the skin after sample processing, leading to false-negative SAA responses in samples exposed to non-ultra-low temperatures or repeated freezing and thawing cycles. The storage condition significantly impacted the number of positive replicates of the SAA response.

To assess potential fluctuations in SAA based on the plate utilized, two black 96-well plates with a clear bottom (Nunc, Thermo Fisher, and Sigma-Aldrich) were used. The amplification buffer containing seeds was prepared at the same time and then split into both the Nunc and polystyrene plates. Eight samples from PD patients and 5 samples from controls were analyzed. Both plates exhibited excellent diagnostic accuracy in distinguishing PD from non-synucleinopathies, achieving a sensitivity and specificity of 100%. Analysis of the entire cohort using SAA revealed a gradual increase in ThT fluorescence in skin biopsies from the PD group overtime on both types of plates used. Specifically, on Nunc plates, the fluorescence surpassed the threshold at an average of 5.78 (±1.06) hours, whereas on polystyrene plates, it occurred at 6.15 (±1.15) h (Fig. 4e). Interestingly, the maximum ThT fluorescence (Fmax) was higher in the Nunc (97.00 ± 2.64%) compared to the polystyrene group (93.01 ± 2.31%) after 12 h of amplification using the modified SAA, although this difference did not reach statistical significance (Fig. 4f). These findings indicate that the Nunc plate, designed with coatings tailored for specific applications such as minimizing background fluorescence in fluorescence assays, is the preferred choice for conducting the SAA experiment.

### Reproducibility of the streamlined SAA assay between different study sites

**Fig. 5:**
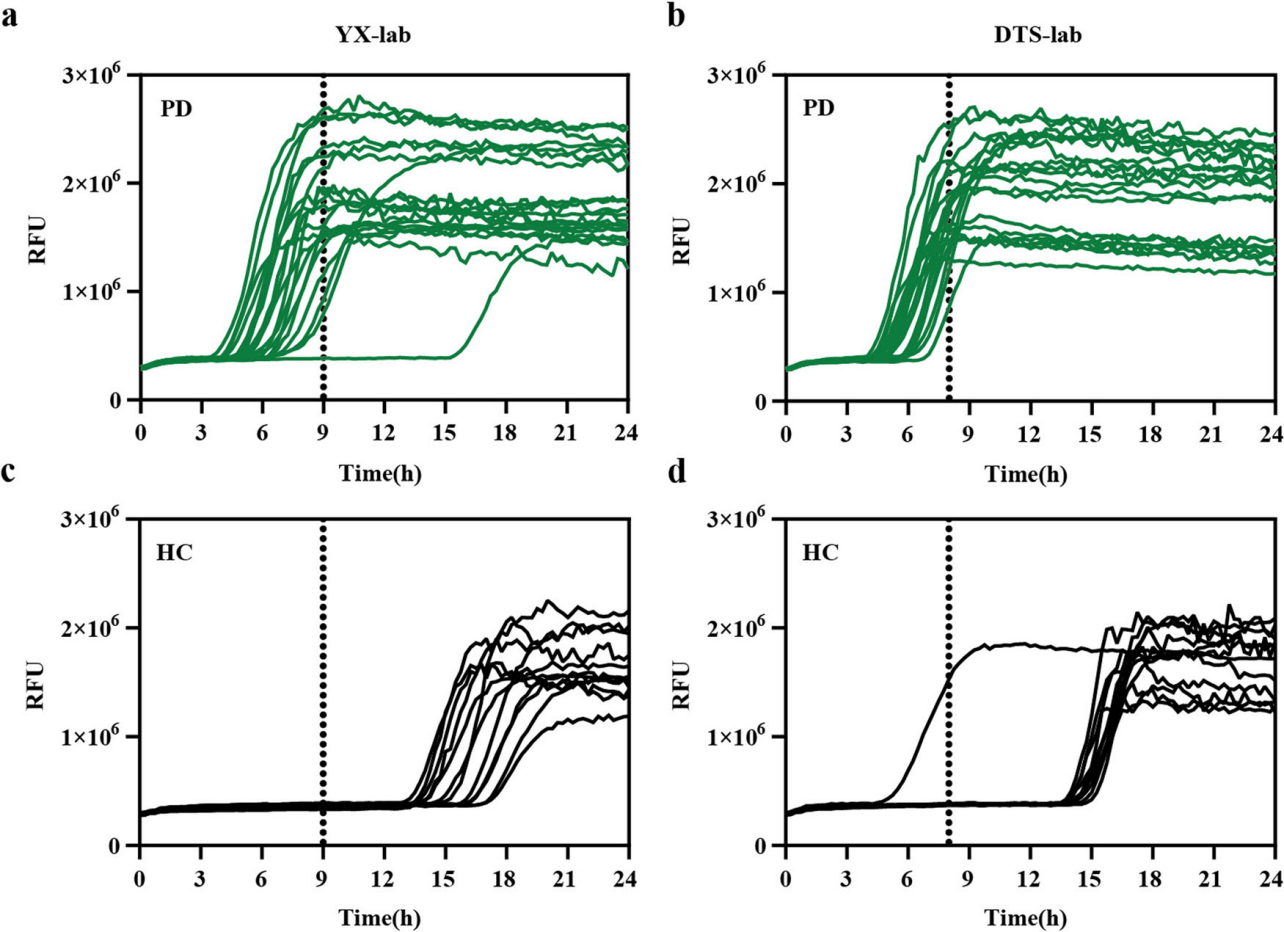
Evaluation of the consistency of streamlined SAA utilizing skin homogenate from PD patients and HC. **a–d** In the streamlined SAA reactions, skin homogenates from both PD patients and HC were subjected to analysis. The seeding activity of αSynD was detected in all 20/20 skin samples from PD patients at DTS-lab and 19/20 of the same PD patients at YX-lab (**a** and **b**). Conversely, none of the samples from HC induced seeding activity at YX-lab, while 1/12 skin samples from HC exhibited seeding activity at DTS-lab (**c** and **d**). The curves depicted in the graphs were generated by plotting the average fluorescence intensities of each sample over time.

After confirming the efficacy of the simplified αSyn SAA protocol with skin samples from individuals with PD and non-neurodegenerative controls, we proceeded to assess the inter-laboratory reproducibility of the streamlined assay. To gauge this reproducibility, trials were initiated in DTS-lab and YX-lab blindly utilizing skin samples collected from 20 individuals with PD and 12 individuals with controls. The demographic data and the αSyn SAA results are reported in Table 2. Figure 5a–d illustrates graphical representations of the seeding activities observed in skin samples from both laboratories. Utilizing lab-specific time thresholds depicted in the method, we noted that the skin samples from the same PD patients (19 out of 20) consistently exhibited seeding activity, resulting in a 95% interlaboratory reproducibility. For control samples, none induced seeding activity, except for one sample at YX-lab, leading to a 91.7% inter-laboratory reproducibility between the two facilities. Overall, the data demonstrated a 93.8% inter-laboratory reproducibility between DTS-lab and YX-lab. Consequently, the optimized αSyn SAA showcased significant agreement between raters (Kappa = 0.87, 95% CI 0.69–1.00)(Table 2).

**Table 2:** Comparison of streamlined αSyn SAA results between DTS-lab and YX-lab

	PD		HC	
	YX-lab	DTS-lab	YX-lab	DTS-lab
Samples analyzed	20	20	12	12
αSyn’s seeding activity	19	20	0	1
Meantime at fluorescence threshold (SD)	7.6 (1.1)	6.8 (1.3)	NA	NA
Seeding activity vs. clinical diagnosis (sensitivity)	95.0%	100%	0	8.3%
Disease-specific interrater agreement between labs	95.0%		91.7%	
Overall interrater agreement between labs	93.8% (Kappa = 0.87, 95% Cl 0.69–1.00)

## Discussion

αSyn SAA has emerged as a valuable diagnostic tool, detecting abnormal aggregates of αSyn associated with PD and other synucleinopathies. Various research groups have demonstrated its initial applications using brain homogenates, olfactory mucosa, saliva, and skin^17,24–26^. This involves detecting the seeding activity of αSyn, capitalizing on the intrinsic ability of amyloid fibrils to self-template^21,26–32^. However, current SAA protocols exhibit differences in configuration, assay duration, tissue processing methods, and the sources of reagents. Despite the commitment of multiple research groups to optimizing assay conditions to enhance the likelihood of routinely detecting low levels of seeds in tissues^33,34^ and to cut down the time required^35,36^. In our research, we demonstrated the customization of physicochemical factors governing the in vitro amplification of αSyn to enhance its elongation and improve the efficiency of SAA. Using a screening approach, we have generated a streamlined SAA assay that allows us to detect αSynD utilizing skin samples in less than 24 h. More importantly, we explore the whole process of pretreatment, storage as well as amplification utilizing skin samples. It showed this streamlined assay can discriminate between PD patients and controls with sensitivity, specificity, as well as reproducibility, resulting in a simplified assay platform that can be easily implemented in a routine laboratory or clinical application.

Various research teams globally are adopting and validating αSyn SAA on human skin under diverse conditions^8,12,26,37,38^. However, the assay faces limitations related to sensitivity to sample quality, resource intensity, and variable standardization across various laboratories. To achieve an accurate diagnostic value in a more accessible way, we systematically assessed 150 different reaction conditions and optimized the method through three steps: elevating the incubation temperature to 50 °C, utilizing mouse-derived instead of human-derived monomeric αSyn, and incorporating 10% AS into the incubation buffer. Our data unequivocally show that selecting the appropriate conditions for seed amplification is highly significant. As is reported, other groups have also extensively evaluated SAA conditions for αSynD testing, such as temperature, SDS concentration, silica beads, pH, or the presence of anions and cation^35,39–41^. However, there has been no prior exploration of tissue-specific, particularly skin-specific buffers to enhance our comprehension of the optimal in vitro physicochemical factors conducive to the detection of specific seed conformers. Intact αSyn fibrils that can be extracted from Lewy bodies are sarkosyl-insoluble^42,43^, however, there are limited reports on its application for extracting αSyn from skin tissues. This disparity could stem from the fact that αSyn primarily forms mature insoluble aggregates known as “amyloid fibrils” in the brain^44^. In contrast, αSyn aggregates in the skin may manifest in diverse forms such as linear fibers or granules, potentially differing in morphology and structure from brain aggregates^12,45^. This distinction underscores the complexity of αSyn aggregation across different tissues and necessitates further exploration to elucidate the underlying implications. A deeper insight into how tissue-specific physicochemical factors influence the aggregation of various αSyn forms will contribute to the advancement of rapid and sensitive in vitro assays for the diagnosis of PD and other synucleinopathies.

Our approach enables a notable decrease in the experiment duration. The modified conditions present a practical approach for swiftly detecting αSyn seeding activity, achieving results in less than 24 h in skin samples from PD patients without compromising diagnostic accuracy or specificity^12,26,38^. In contrast, the amplification phase for SAA typically exceeds 50 h under buffer conditions^8,12,26^. However, it is important to note that some published protocols have a very similar “speed” of execution compared to our method^35,37,38^. In these protocols, most positive samples cross the threshold between 9 and 20 h, with virtually all crossing before 24 h. Additionally, these protocols have been found to be very robust, having been tested against atypical parkinsonism and in a large cohort of iRBD patients. Furthermore, with such protocols, none of the negative samples reach the threshold during the observation period. This aspect is a clear advantage for accurately distinguishing between negative and positive samples. Besides the decreased reaction time, our modified conditions attained a detection sensitivity of about 100 fg, a level of performance comparable to that of conventional αSyn SAA in terms of sensitivity^26,46^, and thus it can be used to detect skin αSyn aggregates at early stages of PD. In this domain, employing suboptimal conditions could result in the absence of seeding activity, indicating a potential absence of seeds. However, it might also signify the incapacity of the specific SAA conditions to facilitate the amplification of those specific seeds.

The mechanistic reasons for the improvements observed with the streamlined SAA are not clear and likely involve complex factors. As the elevated temperature consistently provided more reaction momentum conducive to αSyn aggregation, it is reasonable to suggest that intermittent shaking at 50 °C can increase the collision rate of αSynD, effectively shortening the recruitment time of monomers at the end of fibrils. Regarding the substrate, our data indicated that MM allowed for much faster PD skin-seeded reactions, but only when the reaction mixture was supplemented with AS at 50 °C. As highlighted by previous experts, the amino acid sequences of the mouse and human proteins differ at seven positions, particularly at A53T, which adopts a “natively unfolded” or disordered structure. The mouse protein forms amyloid fibrils more rapidly than its human counterpart, resulting in a significantly shorter lag phase compared to human WT, A53T, or A30P^47,48^. Anions, particularly SO4^−^, induce the partial folding of αSyn at neutral pH, giving rise to a crucial protein–water–anion interaction. In turn, this leads to a rapid amplification of oligomeric/multimeric αSyn seeds^33,40^. Paradoxically, adding high levels of AS to reaction mixtures had a negative impact on the speed and intensity of PD skin-seeded reactions at all tested temperatures. Therefore, there seems to be a synergistic beneficial effect of including AS and using MM as a substrate at 50 °C. However, the specific ratio of each substrate also holds significance in experimental setups. Besides the impact of the buffer condition on amplification, the quality of the sample itself significantly influences the outcome. Unlike the loosely structured brain tissue, the dermis sub-layer appears denser, contains fewer cells, and has thicker collagen bundles. Consequently, more intense grinding is required for adequate protein exposure. According to our findings, excessive grinding made αSyn aggregates prone to depolymerization. Therefore, appropriate grinding could bring out the intrinsic properties of self-templates of αSynD.

The effect of grinding on protein integrity is a nuanced topic that depends on various factors, including the type of protein, grinding conditions, and intended applications^49^. Here are some key points to consider. On the one hand, grinding involves mechanical forces that can cause stress on proteins^50^. This stress may lead to the unfolding or denaturation of the protein, disrupting its native conformation and potentially affecting its functional properties. On the other hand, grinding can promote protein aggregation^51^. This can occur due to the exposure of hydrophobic regions or the creation of new interaction interfaces during the grinding process. In our experimental setup, the control experiment involving grinding monomers alone did not exhibit any seeding activity. However, we cannot discount the potential for grinding to trigger αSyn aggregation when both monomers and PFFs are present simultaneously. This could explain the observed retention of seeding activity on the surface. Therefore, additional experiments are warranted to clarify the underlying mechanism.

Previous studies have announced that more than half of αSyn-positive patients with iRBD converted to PD or DLB after years of follow-up^21^. In patients with iRBD, our findings showed that the modified SAA identifies misfolded αSyn in skin samples with a sensitivity of 66.6%, which was slightly lower than those found previously in human cerebrospinal fluid. Previous studies from other groups have shown that not all the patients with iRBD will ultimately end up with αSyn disorders^52,53^, which may contribute to the low sensitivity of αSyn aggregates detection in iRBD by in vitro amplification. A longitudinal evaluation is required in future studies. Anyway, this may indicate the positive predictive values of our method for patients with iRBD and an undefined diagnosis of parkinsonism.

Collectively, these results demonstrate a new skin-based αSyn amplification assay which provides a more rapid diagnostic, with comparable sensitivity and specificity in distinguishing PD cases from controls when compared to conventional SAA assays. Given the less invasive nature of skin punch biopsy, the seeding activity of skin αSyn may be a practical preclinical diagnostic biomarker for PD and may be far in advance of neuronal damage and clinical signs of synucleinopathies.

## Methods

### Expression and purification of recombinant human/mouse αSyn Monomer

Purification of human/mouse full-length αSyn (1–140 amino acid) was performed as described previously with minor modifications^54^. In summary, the BL21 (DE3) *E. coli* strain (TransGen, Beijing, China) was transformed with a plasmid expressing full-length SNCA/Snca. The culture medium was agitated at 230 rpm overnight until the optical density reached 0.8 at 600 nm, followed by induction with 1.0 mM isopropyl β-d-thiogalactoside for 4 h. After centrifugation at 8000 × g for ten minutes at 4 °C, the bacterial pellets were resuspended in a high salt buffer comprising 10 mM Tris–HCl (pH 7.5), 500 mM NaCl, 1 mM EDTA, 1 mM PMSF, and protease inhibitors, and then sonicated on ice. The lysate was boiled for 10 min, followed by ultracentrifugation (Beckman, Brea, USA) at 20,000×*g* for 30 min at 4 °C. After precipitation with 45% saturated ammonium sulfate, the proteins were resuspended in a buffer containing 10 mM Tris–HCl, pH 7.5, and 50 mM NaCl. The solution’s pH was adjusted to 3.0 using HCl. Proteins were removed by centrifugation, and the pH was then readjusted to 7.5 with NaOH. Subsequently, the proteins were purified using a HiTrap column (GE Healthcare, PA, USA) and dialyzed in 10 mM Tris-buffer (pH 7.5) containing 50 mM NaCl before filtration through a 100-kDa Amicon Ultra filter (Millipore, MA, USA).

### Generation of αSyn PFFs from recombinant WT αSyn

Purified HM and MM were used to generate αSyn hPFFs and mPFFs according to the previous protocol^55^. HM and MM, purified separately, were diluted in 10 mM Tris (pH 7.5) containing 50 mM NaCl. Subsequently, they underwent shaking (Eppendorf, Hamburg, Germany) at 1000 rpm for consecutive 7 days to generate αSyn PFFs. Following this, the αSyn PFFs underwent probe-sonication at 20% power, with a total of 60 pulses (one second on and one second off). The concentration levels of endotoxin in αSyn monomers and PFFs were determined using an Endotoxin Detection kit (Bioendo Technology, Xiamen, China). The PFFs were kept in a solution of 10 mM Tris (pH 7.5) with 50 mM NaCl at −80 °C, and the recommended storage concentration is 5 mg/ml, following standard PFFs handling protocols^55^.

### Transmission electron microscopy (TEM)

A 10 μL aliquot of the samples was meticulously deposited onto a TEM grid, which had been previously loaded onto a freshly glow-discharged 400 mesh carbon-coated copper grid (Electron Microscopy Sciences, Pennsylvania, USA). The grid was allowed to incubate for two minutes. After this incubation period, the grid underwent a quick rinse with three drops of a 50 mM Tris-HCl solution at a pH of 7.5. Subsequently, the grid was consistently floated on two drops of a 0.75% uranium formate solution (Solarbio, Beijing, China). Visualization of the grids was carried out using a CM 120 transmission electron microscope (PHILIPS, Eindhoven, Netherlands), operating at an acceleration voltage ranging between 80-120 kV. This methodology facilitated a detailed examination of the specimens.

### Primary neuron culture and αSyn PFFs Treatments

For the preparation of primary neurons, pregnant C57BL/6 mice (Southern Medical University, Guangzhou, China) were sacrificed by CO_2_ inhalation at embryonic day 17. Embryos were dissected, and the cortex was trypsinized and dissociated using a fire-polished Pasteur pipette as mentioned previously^56^. Cells were seeded on poly-L-ornithine/laminin-coated plates (Sigma-Aldrich, St. Louis, MO, USA) at a density of 500,000/cm^2^ containing neurobasal medium (Thermofisher, CA, USA) with additional penicillin/streptomycin (Gibco, Darmstadt, Germany), L-glutamine (Seromed, Dollnstein, Germany), and B27 supplement (Thermofisher, CA, USA). On the 5th day after seeding on the plates, cells were treated with pretreated or freshly prepared hPFFs (1 μg/ml) for 10 days.

### Immunofluorescence

Primary neurons for immunofluorescence (IF) staining were cultured on coverslips under specific experimental conditions. Skin tissue samples were sliced into 10 µm sections and fixed using a 4% paraformaldehyde solution. These fixed samples were then treated with a PBS solution containing 0.01% Triton X-100 and 5% BSA for 1 h at RT. Subsequently, the cells or skin slides were incubated overnight at 4 °C with the primary antibody anti-p-syn (pS129 αSyn) (CST, No. 51253, USA, diluted at 1:500). Slides were washed three times with PBS and then incubated with Alexa Fluor 488-labeled secondary antibodies (Thermofisher, CA, USA) at a dilution of 1:500 for 1 h at RT. Fluorescence images were captured using a NIKON ECLIPSE 3000 fluorescence microscope (Tokyo, Japan). Phosphorylated αSyn was detected using pS129-Syn, and DAPI was used to identify nuclei. Each experimental condition was repeated in three independent experiments.

### Pretreatment of PFFs

For grinding the PFFs, 500 μl of h/mPFFs (1 μg/ml) were mixed with one 5 mm and four 2 mm zirconium oxide beads (Thermofisher, California, USA) in a 1.5 ml EP polypropylene tube. The tube was then shaken at a frequency of 75 Hz for 45 s in a homogenizer (Luka, Guangzhou, China). This process was repeated for a total of 1–10 cycles of vibration grinding. To evaluate whether monomeric αSyn could alleviate the reduction in seeding activity induced by grinding, 0.1 mg/ml of monomeric αSyn was added to the h/mPFFs before grinding, and the same concentration of monomeric αSyn was used as control, with both subjected to 10 cycles of vibration grinding.

### Skin biopsy procedure and sample preparation

A total of 617 biopsy skin samples were collected from the Department of Neurology at the First Affiliated Hospital of Guangzhou Medical University between 2016 and 2022. In our experimental setup, every participant underwent a thorough clinical research assessment. This included evaluating their cognitive and motor functions by conducting a mental state examination using the Mini-Mental State Exam, carrying out a structured physical neurological examination, particularly focusing on motor assessments using the Hoehn and Yahr scale and the Unified Parkinson’s Disease Rating Scale (UPDRS) Part III motor assessment. All participants were enrolled in a research protocol that allowed for yearly follow-up assessments, with each participant receiving at least one follow-up assessment after their initial visit. Neuroimaging results (MRI, and occasionally FDG PET scans or DaTscan) were reviewed whenever they were available. The diagnosis for each group was determined by movement disorder specialists at the respective sites and later verified by a central consensus committee. αSyn SAA results were not accessible to investigators or the consensus committee during the diagnosis process and therefore, were not considered in the classification of participants. Diagnostic criteria followed established norms: control subjects exhibited no history of parkinsonism and synucleinopathy signs. These samples included 332 subjects with PD who received a clinical diagnosis of probable PD based on the international diagnostic criteria^57^. 28 with progressive supranuclear palsy (PSP), 100 with Essential Tremor (ET), 32 with Alzheimer’s disease (AD), 42 with epilepsy, 6 with viral encephalitis (VE), and 77 HC employing the most recent recommended criteria for AD, PSP, ET, and VE^58–60^. Experienced raters diagnosed rapid eye movement sleep behavior disorder using video polysomnography according to established criteria^61^. Ethical acquisition of biopsy tissues from the skin of consenting patients was carried out, with approval from the Ethics Committee of the First Affiliated Hospital of Guangzhou Medical University (MRE. No.155). Written informed consent was obtained from patients or legal representatives. Using a ring drill extractor (USHIO, Tokyo, Japan), we conducted a three-millimeter punch biopsy taken from a proximal location (C7 paravertebral: 5 cm from the midline). Multiple studies have reported a significant proximal-distal gradient of pathological αSyn in patients with PD, demonstrating a spine gradient with the cervical site expressing the highest positivity^62,63^.

The obtained biopsy specimens were bisected perpendicularly, and both halves were assigned blind codes before being stored at −80 °C. Prior to further processing, the epidermal layer without pathological αSyn deposition was removed, followed by three washes in Tris-buffered saline (TBS). Subsequently, the samples were homogenized using a Luka homogenizer (Guangzhou, China) equipped with one 5 mm and four 2 mm zirconium oxide beads (Thermo Fisher, California, USA). The homogenization was carried out in 500 μl of grinding solution buffer, comprising 10 mM Tris–HCl, 50 mM NaCl, and 1x Proteinase Inhibitor Cocktail, adjusted to pH 7.5. The homogenization process was conducted at a speed of 75 Hz for 45 s and repeated three times to ensure thorough disruption and mixing of the tissue. Subsequently, the homogenized samples were centrifuged at 5000 × *g* for 5 min to separate the cellular debris and larger particles. The resulting supernatant containing the extracted components was either used directly for downstream assays or stored at −80 °C for future analysis.

### Streamlined SAA

1 µl of the skin lysate was added to the reaction mixture containing 0.1 mg/ml mouse αSyn monomer, 10% AS, 10 mM ThT, 50 mM NaCl, and 10 mM Tris (pH 7.5) for a total volume of 100 µl per well of a black 96-well plate with a clear bottom (Thermofisher, CA, USA). Three replicate reactions were made for each skin sample. The plate was sealed with sealing film (Thermofisher, CA, USA) and incubated at 50 °C in a SpectraMax iD5 plate reader (MD, CA, USA) with intermittent shaking (high speed, double orbital, shake for 14 min followed by 1 min pause) throughout the assay. ThT fluorescence (450 ± 10 nm excitation and 490 ± 10 nm emission; bottom read) was recorded every 15 min for a total run of 24 h. A sample was considered positive when at least two out of three replicates crossed the threshold of background fluorescence plus 10 standard deviations (yielding a ~11.6% cut-off threshold) within 8.375 h.

### Perform a reproducibility test in two separate laboratories

Apart from the primary experiment carried out at the First Affiliated Hospital of Guangzhou Medical University in Datansha (DTS-lab), a group of 20 well-characterized patients from the Parkinson and Movement Disorders Unit of DTS-lab (57% male, mean age 68 years, mean disease duration 4.7 years) and a control group of 12 non-neurodegenerative individuals (43% male, mean age 62 years) were included. These samples were analyzed at the Guangzhou Medical University in Yuexiu (YX-lab) using the other half of the biopsy to evaluate the reproducibility of the modified SAA, following a similar assay procedure as previously described. Due to the observed variation in the aggregation rate of rec-αSyn between DTS-lab and YX, we implemented lab-specific time thresholds. A sample was considered capable of inducing seeding activity if at least two out of three replicates exceeded the fluorescence threshold before 9 h at YX-lab and within 8.375 h at DTS-lab. Upon meeting this criterion, the sample was designated as positive (+). The introduction has been corrected in the revised manuscript.

### ^18^F-AV133 PET and CT image processing

The radiotracer [^18^F]-DTBZ (^18^F-AV133) was synthesized using aqueous [^18^F]-fluoride. Subjects received intravenous administration of D6-[^18^F] FP-(+)-DTBZ at a dose of 10.0 mCi ± 10% and then rested for 55 min in a quiet, light-free environment. Brain scans were conducted over a period of approximately 60–70 min following the dynamic drug administration. Florbenazine images were subjected to spatial normalization using an ^18^F-AV133 PET template aligned to a standard atlas. Atlas-based volumes of interest were employed for specific target areas, including the caudate, anterior and posterior putamen, and total striatum. The standard uptake value ratio was calculated by determining the ratio of tracer activity in the target volumes of interest relative to the occipital cortex, serving as the reference region.

### Statistical analysis

All statistical analysis was conducted with GraphPad Prism Software (GraphPad Software Inc., CA, USA). Experimental data were analyzed using the two-tailed independent Student’s *t*-test for comparing two groups and one-way analysis of variance (ANOVA) followed by performing Tukey’s post-hoc-test for three or more groups. Unless otherwise stated, data are presented as mean values with SD. A *P* value of <0.05 was considered statistically significant.

### Supplementary information

Supplementary information

## Data Availability

The datasets generated and analyzed during the current study are available from the corresponding authors upon request.
