# Supplementary material for: A skin-specific α-Synuclein seeding amplification assay for diagnosing Parkinson’s disease
Source: NPJ Parkinsons Dis. 2024 Jul 4;10:129. doi: 10.1038/s41531-024-00738-7 (PMC11222486; doi:10.1038/s41531-024-00738-7)
Supplement: Supplementary file 1 — Supplementary information [file 41531_2024_738_MOESM1_ESM.pdf]

Supplementary Figure 1

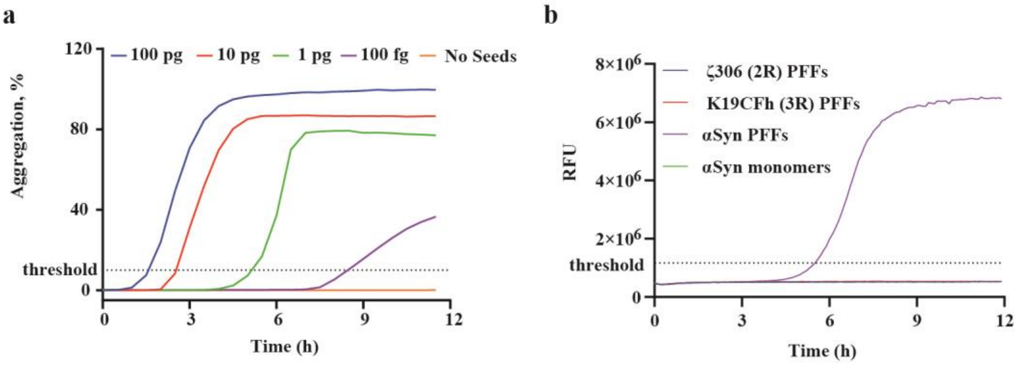

Supplementary Figure 1 Detection limit of hPFFs and response of Tau PFFs in streamlined αSyn-SAA

a. Serial dilutions of hPFFs, corresponding to concentrations of 100 pg, 10 pg, 1 pg, and 100 fg of hPFFs, were introduced into the streamlined αSyn-SAA, with no seed group as control.

b. Homogeneous or diverse seed types, specifically 100 pg of ζ306 (2R) PFFs, 100 pg of K19CFh (3R) PFFs, 100 fg of αSyn hPFFs, and 100 fg of αSyn monomer, were introduced into streamlined αSyn-SAA. The results are expressed as the representative data (n=4).

Supplementary Table 1 Compare the streamlined αSyn SAA and IF in skin samples

	**PD (n=12)**	**non-PD Controls (n=8)**
pS129 [positive, negative]	11 (1)	0 (8)
streamlined SAA [positive, negative]	12 (0)	0 (8)
Interrater agreement	95.0% (Kappa=0.898, 95% Cl 0.70-1.00)	
